# Supplementary material for: Cross-sectional evaluation of the multidimensional indicators of psychosocial functioning and its sociodemographic correlates among Indian adults: WHO SAGE Study (2007–2010)
Source: PLOS Glob Public Health. 2024 Apr 25;4(4):e0003102. doi: 10.1371/journal.pgph.0003102 (PMC11045086; doi:10.1371/journal.pgph.0003102)
Supplement: S1 Checklist — (DOCX) [file pgph.0003102.s001.docx]

STROBE Statement—checklist of items that should be included in reports of observational studies

|  | Item No. | Recommendation | Page  No. | Relevant text from manuscript |
| --- | --- | --- | --- | --- |
| **Title and abstract** | 1 | (*a*) Indicate the study’s design with a commonly used term in the title or the abstract | Page 15 Analytic strategy | “All analyses in the study were cross-sectional in nature and conducted using STATA version 15.1” |
|  |  | (*b*) Provide in the abstract an informative and balanced summary of what was done and what was found | Page 2 and 3 | This information are stated in the study abstract |
| Introduction | | | |  |
| Background/rationale | 2 | Explain the scientific background and rationale for the investigation being reported | Page 5 | Background and rationale for the study is discussed in introduction and summarized at the end of introduction section “Therefore, the goal of the study is to create a gender-specific evidence-base to describe how PF vary based on sociodemographic correlates among Indian population subgroups” |
| Objectives | 3 | State specific objectives, including any prespecified hypotheses | Page 11 | “This study has two aims. The first aim is to evaluate gender differences in the levels of PF in India. Based on previous research, it is hypothesized that women will have a greater burden of difficulties in their PF than men. The second aim is to assess sociodemographic differences in the levels of PF among Indian men and women. It is hypothesized that experience of social or economic disadvantage will be related to lowered levels of personal and social functioning.” |
| Methods | | | |  |
| Study design | 4 | Present key elements of study design early in the paper | Page 11 | Key elements of study design is written under ‘data and sample’ section of Methods. |
| Setting | 5 | Describe the setting, locations, and relevant dates, including periods of recruitment, exposure, follow-up, and data collection | Page 11 in data and sample | “SAGE is representative of the adult population 18 years and older in six states: Assam, Karnataka, Maharashtra, Rajasthan, Uttar Pradesh, and West Bengal. These states were selected from among Indian states with populations of more than five million based on their geographic region and level of economic and human development.” |
| Participants | 6 | (*a*) *Cohort study*—Give the eligibility criteria, and the sources and methods of selection of participants. Describe methods of follow-up  *Case-control study*—Give the eligibility criteria, and the sources and methods of case ascertainment and control selection. Give the rationale for the choice of cases and controls  *Cross-sectional study*—Give the eligibility criteria, and the sources and methods of selection of participants | Page 11 | “SAGE Wave 1 included a total of 11,230 completed interviews: 4,670 interviews with individuals aged 18-49 (3,625 women and 1,045 men) and 6,560 interviews with individuals aged 50-plus (3,256 women and 3,304 men).” |
|  |  | (*b*) *Cohort study*—For matched studies, give matching criteria and number of exposed and unexposed  *Case-control study*—For matched studies, give matching criteria and the number of controls per case |  |  |
| Variables | 7 | Clearly define all outcomes, exposures, predictors, potential confounders, and effect modifiers. Give diagnostic criteria, if applicable | Page 12 Measures | All variables are defined and discussed under ‘Measures’ subsection. |
| Data sources/ measurement | 8* | For each variable of interest, give sources of data and details of methods of assessment (measurement). Describe comparability of assessment methods if there is more than one group | Page 12 Measures | All variables are defined and discussed under ‘Measures’ subsection. |
| Bias | 9 | Describe any efforts to address potential sources of bias | Page 15 Analytic strategy | All key hypotheses were evaluated in adjusted models with relevant sociodemographic indicators added as controls. We also stratified analysis by gender to reduce bias introduced because of different lived experiences of Indian men and women. “Next, each PF indicator was regressed on gender to evaluate whether these indicators varied by gender after controlling other sociodemographic characteristics (Table 2). Finally, the relationship between sociodemographic characteristics and PF indicators were examined using ordinary least square regression (OLS) models and logistic regression model, separately for men and women (Table 3).PF indicators, including social indicators of interpersonal relationship difficulty, social connectedness, and personal indicators of sleep, affect, perceived quality of life, and cognition were each regressed on sociodemographic factors” |
| Study size | 10 | Explain how the study size was arrived at | Page 11 and 12 | “SAGE Wave 1 included a total of 11,230 completed interviews: 4,670 interviews with individuals aged 18-49 (3,625 women and 1,045 men) and 6,560 interviews with individuals aged 50-plus (3,256 women and 3,304 men).” |

Continued on next page

| Quantitative variables | 11 | Explain how quantitative variables were handled in the analyses. If applicable, describe which groupings were chosen and why | Page 12 - 15 | These are described in details under ‘Measures’ subsection |
| --- | --- | --- | --- | --- |
| Statistical methods | 12 | (*a*) Describe all statistical methods, including those used to control for confounding | Page 15 | “There were three steps in this analysis. First, weighted means and percentages of key study variables were estimated; chi-square tests were used to assess significant men-women differences (Table 1). Next, each PF indicator was regressed on gender to evaluate whether these indicators varied by gender after controlling other sociodemographic characteristics (Table 2). Finally, the relationship between sociodemographic characteristics and PF indicators were examined using ordinary least square regression (OLS) models and logistic regression model, separately for men and women (Table 3). PF indicators, including social indicators of interpersonal relationship difficulty, social connectedness, and personal indicators of sleep, affect, perceived quality of life, and cognition were each regressed on sociodemographic factors.” |
|  |  | (*b*) Describe any methods used to examine subgroups and interactions |  | No interactions were examined |
|  |  | (*c*) Explain how missing data were addressed | Page 16 | “Using Stata, missing data was handled by omitting the row with the missing values.” |
|  |  | (*d*) *Cohort study*—If applicable, explain how loss to follow-up was addressed  *Case-control study*—If applicable, explain how matching of cases and controls was addressed  *Cross-sectional study*—If applicable, describe analytical methods taking account of sampling strategy | Page 16 | “To account for the multi-stage sampling design of the study, survey weights were applied throughout” |
|  |  | (*e*) Describe any sensitivity analyses |  | Non applicable |
| Results | | | | |
| Participants | 13* | (a) Report numbers of individuals at each stage of study—eg numbers potentially eligible, examined for eligibility, confirmed eligible, included in the study, completing follow-up, and analysed |  | Not applicable |
|  |  | (b) Give reasons for non-participation at each stage |  | Not applicable |
|  |  | (c) Consider use of a flow diagram |  | Not applicable |
| Descriptive data | 14* | (a) Give characteristics of study participants (eg demographic, clinical, social) and information on exposures and potential confounders | Page 16 | Listed in Table 1 and described in text:  Table 1 shows the sample descriptive characteristics by gender. Majority of the sample belonged to early adulthood age category (26-45 years; 52.3%). Among men, 27.1% reported having completed less than primary school education (less than 5 years of school); however, 48.7% of the women reported no formal education. A majority of the sample belonged to the ‘General caste’ category (60%) and reported being currently married (81.7%). Women were more likely to report being widowed (13.1%) than men (3.3%; *p* < .0001). A majority of the sample also reported being Hindu (84.2%) and living in an urban area (74.3%). Men (76.4%) were more likely to live in urban areas than women (72.2%; *p* = .007). Most of the men reported being ‘self-employed’ (48.3%) followed by informal employment (26.9%), whereas a majority of women reported ‘not working’ (55.4%) followed by informal employment (19.9%). There were no significant gender differences based on income quintiles and religion. |
|  |  | (b) Indicate number of participants with missing data for each variable of interest |  |  |
|  |  | (c) *Cohort study*—Summarise follow-up time (eg, average and total amount) |  |  |
| Outcome data | 15* | *Cohort study*—Report numbers of outcome events or summary measures over time |  |  |
|  |  | *Case-control study—*Report numbers in each exposure category, or summary measures of exposure |  |  |
|  |  | *Cross-sectional study—*Report numbers of outcome events or summary measures | Page 19 | Described in Table 3 |
| Main results | 16 | (*a*) Give unadjusted estimates and, if applicable, confounder-adjusted estimates and their precision (eg, 95% confidence interval). Make clear which confounders were adjusted for and why they were included | Page 18 | See Table 2 |
|  |  | (*b*) Report category boundaries when continuous variables were categorized |  |  |
|  |  | (*c*) If relevant, consider translating estimates of relative risk into absolute risk for a meaningful time period |  | Not applicable |

Continued on next page

| Other analyses | 17 | Report other analyses done—eg analyses of subgroups and interactions, and sensitivity analyses |  |  |
| --- | --- | --- | --- | --- |
| Discussion | | | | |
| Key results | 18 | Summarise key results with reference to study objectives | Page 22. Start of discussion. | “Overall, the study found support for these theory-driven key hypotheses – there were significant sociodemographic differences in PF among Indian adults. As such, social or economic disadvantage (in terms of age, income, education, employment status, marital status, and religion, caste) was associated with poorer PF. However, the study observed that socioeconomic patterns in PF were much more nuanced among women than among men. There were also interesting patterns created by some types of sociodemographic indicators that were inconsistent with the theory-based expectation, namely caste, and religion.” |
| Limitations | 19 | Discuss limitations of the study, taking into account sources of potential bias or imprecision. Discuss both direction and magnitude of any potential bias | Page 29 - 30 | “Three important caveats limit the conclusions of the study. First, gender is reported as a binary variable (male and female) in WHO SAGE Wave 1. This restricts the generalizability of the findings to include all categories of gender and sexual identities. Second, the cross-sectional nature of the study precludes causal conclusions. However, prior research and theory suggests that social structures, social hierarchy, and social conditions shape individuals’ life experiences, opportunities and accessibility of resources that perpetuate disparities in functioning and wellbeing. Future research should evaluate changes in sociodemographic characteristics, such as social mobility, and it impact on PF, using longitudinal data. Finally, health behaviors are an important mechanism through which sociodemographic factors create health impacts. However, behaviors are not included in the study. Future studies should evaluate whether health behaviors, such as physical activity, diet, and substance use influence the relationship between sociodemographic variables and PF among these populations” |
| Interpretation | 20 | Give a cautious overall interpretation of results considering objectives, limitations, multiplicity of analyses, results from similar studies, and other relevant evidence | Page 30 | Discussed in conclusion and implications |
| Generalisability | 21 | Discuss the generalisability (external validity) of the study results | Page 29 | “First, gender is reported as a binary variable (male and female) in WHO SAGE Wave 1. This restricts the generalizability of the findings to include all categories of gender and sexual identities. Second, the cross-sectional nature of the study precludes causal conclusions” |
| Other information | |  | | |
| Funding | 22 | Give the source of funding and the role of the funders for the present study and, if applicable, for the original study on which the present article is based |  | Not applicable |

*Give information separately for cases and controls in case-control studies and, if applicable, for exposed and unexposed groups in cohort and cross-sectional studies.

**Note:** An Explanation and Elaboration article discusses each checklist item and gives methodological background and published examples of transparent reporting. The STROBE checklist is best used in conjunction with this article (freely available on the Web sites of PLoS Medicine at http://www.plosmedicine.org/, Annals of Internal Medicine at http://www.annals.org/, and Epidemiology at http://www.epidem.com/). Information on the STROBE Initiative is available at www.strobe-statement.org.
