# Supplementary material for: Cross-sectional evaluation of the multidimensional indicators of psychosocial functioning and its sociodemographic correlates among Indian adults: WHO SAGE Study (2007–2010)
Source: PLOS Glob Public Health. 2024 Apr 25;4(4):e0003102. doi: 10.1371/journal.pgph.0003102 (PMC11045086; doi:10.1371/journal.pgph.0003102)
Supplement: Attachment — Submitted filename: Response to Reviewers.docx [file pgph.0003102.s003.docx]

Daniel Kim, M.D., Dr.P.H.
Academic Editor
PLOS Global Public Health

Subject: Response to Reviewer Comments [GPH-D-23-01132]

Date: November 28, 2023

(GPH-D-23-01132) Cross-sectional evaluation of the multidimensional indicators of psychosocial functioning and its sociodemographic correlates among Indian adults: WHO SAGE Study (2007-2010)

Dear Dr. Kim,

We sincerely appreciate the time and effort invested by you and the reviewers in thoroughly evaluating our manuscript titled ‘Cross-sectional evaluation of the multidimensional indicators of psychosocial functioning and its sociodemographic correlates among Indian adults: WHO SAGE Study (2007-2010).’ Their constructive feedback has been invaluable in enhancing the quality of our work. We have carefully considered each comment and made the necessary revisions to address the concerns raised.

1. **Relevance of the Findings (Comment #1):**

We acknowledge the reviewer's concern regarding the relevance of our study conducted nearly 13 years ago. In response, we have added the following text to the discussion section:

> "Third, the data was collected over 13 years ago, and given the changes in Indian society in the last decade, it would be beneficial to update the findings with the latest data from WHO SAGE Wave 2 India data (2015), which has been made available recently. Meanwhile, the findings from this study can still provide valuable insights into the relationship between psychosocial functioning and sociodemographic characteristics."

2. **Manuscript Headings (Comment #2):**

We appreciate the suggestion to align the headings with the journal norms. Accordingly, we have replaced 'Background' with 'Gaps in Literature' under the 'Introduction' section.

3. **Correction in Text (Comment #3):**

Thank you for pointing out the error in lines 290-291 related to the 'sleep' heading. We have corrected the passage accordingly.

4. **Inclusion of Chi-Square Statistics (Comment #4):**

We acknowledge the suggestion to include chi-square statistics along with p-values in the table. This has been addressed, and the revised tables now include the requested information.

5. **Multivariable Logistic Regression Analysis Goodness of fit (Comment #5):**

We appreciate the guidance on providing more details about the goodness of fit for multivariable logistic regression analysis. To address this, we have included information on model fitness in both the methods and results sections.

In Methods:

> "To evaluate the performance of the multivariate logistic models, we assessed the goodness-of-fit using the Hosmer-Lemeshow tests and also examined the discriminative capability by calculating the area under the receiver operating characteristic (ROC) curve."

In Results:

> "We also assessed the goodness of fit of these models. Overall, both models, for men and women, demonstrated overall goodness of fit as indicated by the Hosmer-Lemeshow test, suggesting no significant discrepancies between observed and expected outcomes across all strata. The overall discriminatory capability of the model, as measured by the adjusted ROC curve, was 0.65 for women and 0.63 for men."

While Stata does not provide R-squared values for weighted logistic analysis of survey data, we have provided alternative measures of model fit.

6. **Stub Headings in Tables (Comment #6):**

We acknowledge the suggestion to include stub headings in Tables 1, 2, and 3. This oversight has been rectified, and stub headings have been added to all tables.

**Comments on the PDF version of the manuscript:**

1. **Comment regarding inclusion of aims in the introduction.**

We have added following sentence at the end of the introduction section

“The aim of this study is twofold: first, to examine gender disparities in psychosocial functioning (PF) among the Indian population; and second, to investigate how PF is associated with various sociodemographic factors within distinct subgroups of the Indian population.”

2. **Interpretation for coefficients and ORs**:

Thank you for the feedback! We have updated the results section with more in-depth details on coefficients and variation explained for each model. We aimed for conciseness while ensuring adequate detail.

3. **Recommendation in discussion of caste and religion**:

Thank you for the feedback. We have discussed the noteworthy results in discussion section line 564 - 587 and 649-666.

4. **Potential for measurement bias**:

Thank you for drawing our attention to this issue. We have added the following sentence to the discussion of limitations: “We recognize that the potential for measurement error in this study could introduce potential limitations to the findings. However, the instruments used in this study were validated by the WHO SAGE survey team (Kowal et al 2012).”

Once again, we express our gratitude to the reviewers for their insightful comments, which have significantly improved the quality of our manuscript. We believe that the revisions made address the concerns raised and enhance the overall clarity of our findings.

Thank you for your consideration, and we look forward to your guidance on the next steps in the review process.

Sincerely,

Apurva Barve, MA PhD
