# Supplementary material for: Cross-sectional evaluation of the multidimensional indicators of psychosocial functioning and its sociodemographic correlates among Indian adults: WHO SAGE Study (2007–2010)
Source: PLOS Glob Public Health. 2024 Apr 25;4(4):e0003102. doi: 10.1371/journal.pgph.0003102 (PMC11045086; doi:10.1371/journal.pgph.0003102)
Supplement: Attachment — Submitted filename: Response to Reviewers_revision2.docx [file pgph.0003102.s004.docx]

Daniel Kim, M.D., Dr.P.H.
Academic Editor
PLOS Global Public Health

Subject: PLOS Global Public Health Decision: Revision required [PGPH-D-23-01132R1]

(GPH-D-23-01132) PGPH-D-23-01132R1

Cross-sectional evaluation of the multidimensional indicators of psychosocial functioning and its sociodemographic correlates among Indian adults: WHO SAGE Study (2007-2010)

Dear Dr. Kim,

We sincerely appreciate the time and effort invested by you and the reviewers in thoroughly evaluating our manuscript revision titled ‘Cross-sectional evaluation of the multidimensional indicators of psychosocial functioning and its sociodemographic correlates among Indian adults: WHO SAGE Study (2007-2010).’ Your feedback has been invaluable in enhancing the quality of our work. We have carefully considered each comment and made the necessary revisions to address the concerns raised.

- **Feedback regarding reference list**

After carefully cross-checking each cited article against the reference section we found five extra references that were added which were not found as cited in the manuscript body text. As a result, we have removed these extra references without any citations in the manuscript-text. These references as listed below are now deleted from the revision manuscript and also included in the marked revision of the manuscript:

1. Ganguli, Mary, Sanjay Dube, Janet M. Johnston, Rajesh Pandav, Vijay Chandra, and Hiroko H. Dodge. 1999. “Depressive Symptoms, Cognitive Impairment and Functional Impairment in a Rural Elderly Population in India: A Hindi Version of the Geriatric Depression Scale (GDS-H).” International Journal of Geriatric Psychiatry 14(10):807–20. doi: 10.1002/(SICI)1099-1166(199910)14:10<807::AID-GPS31>3.0.CO;2-#.
2. Grover, Sandeep, Alakananda Dutt, and Ajit Avasthi. 2010. “An Overview of Indian Research in Depression.” Indian Journal of Psychiatry 52(7):178. doi: 10.4103/0019-5545.69231.
3. Gupta, Dipankar. 2000. Interrogating Caste: Understanding Hierarchy and Difference in Indian Society. Penguin Books India.
4. Sekine, Michikazu, Tarani Chandola, Pekka Martikainen, Michael Marmot, and Sadanobu Kagamimori. 2006. “Work and Family Characteristics as Determinants of Socioeconomic and Sex Inequalities in Sleep: The Japanese Civil Servants Study.” Sleep 29(2):206–16. doi: 10.1093/sleep/29.2.206.
5. Zhang, Bin, and Yun-Kwok Wing. 2006. “Sex Differences in Insomnia: A Meta-Analysis.” Sleep 29(1):85–93. doi: 10.1093/sleep/29.1.85.

We also added following two references in the reference section as they were found to be missing:

1. Charlson FJ, Ferrari AJ, Santomauro DF, Diminic S, Stockings E, Scott JG, McGrath JJ, Whiteford HA. Global epidemiology and burden of schizophrenia: findings from the global burden of disease study 2016. Schizophrenia bulletin. 2018 Oct 17;44(6):1195-203.
2. Reddy VE. Mental health issues and challenges in India: A review. International Journal of Social Sciences Management and Entrepreneurship (IJSSME). 2019 Nov 21;3(2).

- **Supporting information**

To the best of our knowledge, supporting information added to the manuscript submission was the ‘Storbe Checklist’ and ‘Clinical studies checklist’. We have added these to the revision submission as well.

- **Editor comments regarding dataset**

We acknowledge the concern regarding the relevance of our study conducted nearly 13 years ago. In response, we have added the following text to the discussion section in revision 1:

> "Third, the data was collected over 13 years ago, and given the changes in Indian society in the last decade, it would be beneficial to update the findings with the latest data from WHO SAGE Wave 2 India data (2015), which has been made available recently. Meanwhile, the findings from this study can still provide valuable insights into the relationship between psychosocial functioning and sociodemographic characteristics."

We have also added following sentence to elaborate

> “Future research using newer datasets that highlights differences in health outcomes especially as they relate to gender and other socioeconomic variables such as caste is required.”

Once again, we express our gratitude to the reviewers and editor for their insightful comments, which have significantly improved the quality of our manuscript. We believe that the revisions made address the concerns raised and enhance the overall clarity of our findings.

Thank you for your consideration, and we look forward to your guidance on the next steps in the review process.

Sincerely,

Apurva Barve, MA PhD
